# P-1888. Understanding Infection Control Needs in Nebraska Schools: Insights from School Health Staff

**DOI:** 10.1093/ofid/ofaf695.2057

**Published:** 2026-01-11

**Authors:** Christina Cashatt, Mounica Soma, Juan M Teran Plasencia, M Salman Ashraf, Laura Kate K Tyner, Alice I Sato, Robin M Williams, Andrea M Riley

**Affiliations:** Nebraska ICAP, Papillion, Nebraska; Nebraska Medicine, Omaha, Nebraska; University of Nebraska Medical Center/Division of Infectious Diseases, Omaha, NE; University of Nebraska Medical Center, Omaha, Nebraska; Nebraska Medicine, Omaha, Nebraska; UNMC; Children's Nebraska, OMAHA, Nebraska; NE DHHS, Lincoln, Nebraska; Children's Nebraska, Lincoln, Nebraska

## Abstract

**Background:**

The Nebraska (NE) Infection Control Assessment and Promotion Program (ICAP), supported by the NE DHHS HAI/AR Program via a CDC grant, partnered with Children’s Nebraska and the Global Center for Health Security at UNMC to support K-12 school staff during the COVID-19 pandemic. Various infection prevention and control (IPC) resources were developed, including a newsletter, statewide conference (April 2023), online continuing education, and the development of the Nebraska Infection Control in Education (NICE) Book. Because IPC needs in schools are not well understood, we conducted a learning needs assessment (LNA).

**Table 1:**
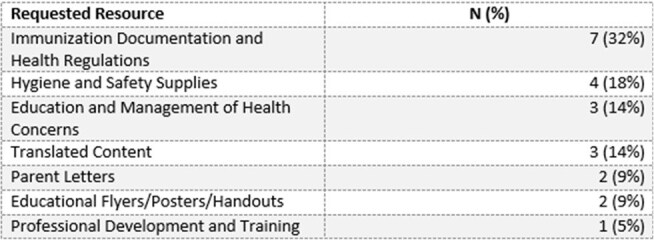
Priority Needs for Additional NICE Book Resources

**Figure 1:**
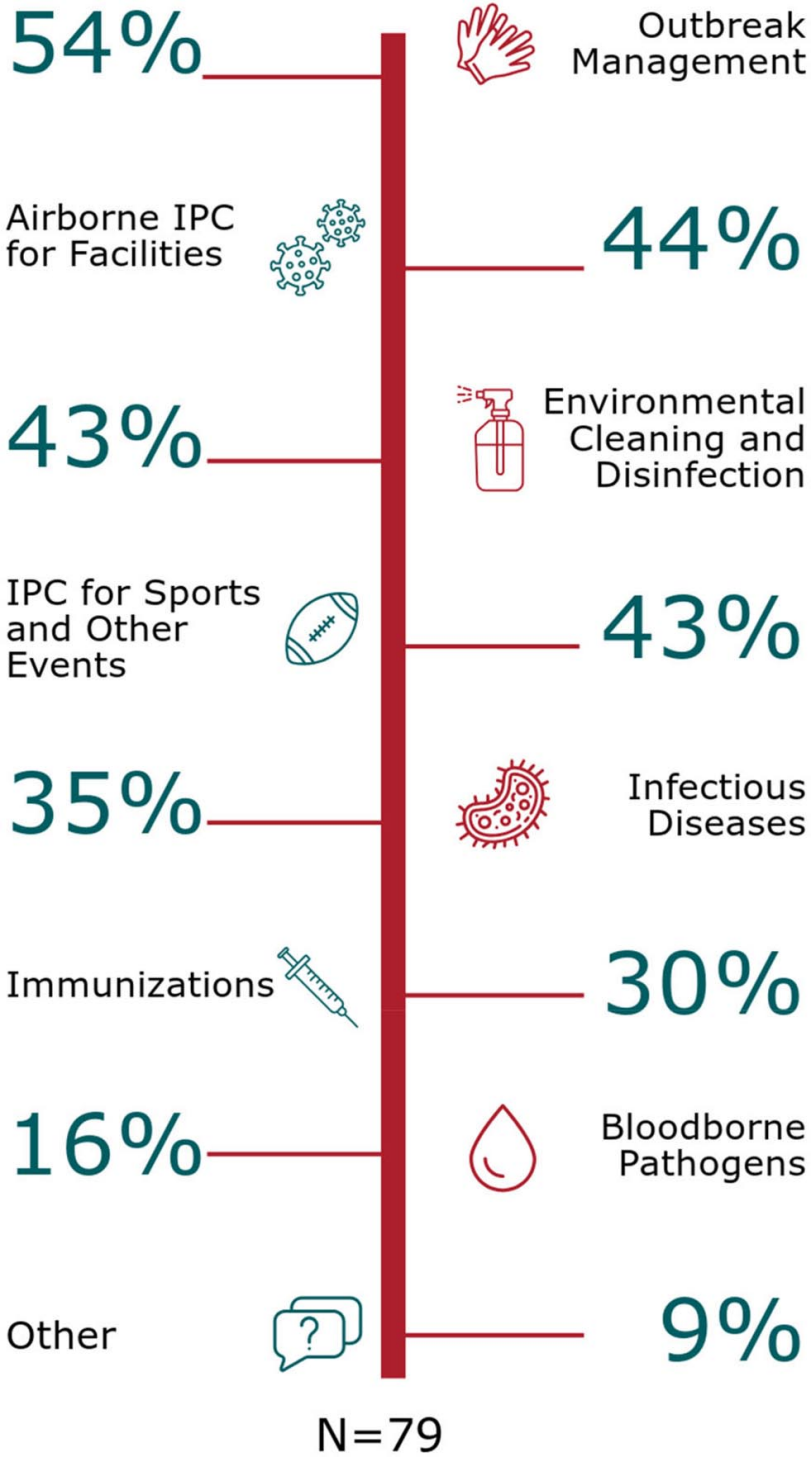
Identified Areas for Additional Support and Education in Infection Prevention and Control

**Methods:**

A REDCap LNA survey was distributed via email to school nurse contact lists, a social media campaign, and the NE-ICAP website from September to October 2024. The survey was directed at school nurses, health paraprofessionals, or designated administrators in healthcare roles. It began with two screening questions to confirm respondents were based in Nebraska and worked in schools. Eligible participants answered ten questions. Descriptive statistics were calculated, and thematic analysis of open-ended responses was performed by subject matter experts.

**Results:**

Of 88 individuals who completed the survey, 9 were excluded based on initial screening, leaving 79 responses for analysis. The NICE book was the most frequently used tool, with 70% reporting use in the past 12 months. For the book, Spanish was the most requested language for resources (78%). The most frequently requested additional topics/tools were *Immunization documentation and health regulation* (32%) (e.g., requirement for vaccines, exemptions, guidance for children entering from outside the U.S), followed by information regarding *hygiene and safety supplies* (18%) (Table 1). The most requested topics for the newsletter were *infectious diseases and common illnesses* (54%), followed by *general IPC topics* (41%). Key areas needing additional education included *outbreak management* (54%) and *airborne disease transmission prevention* (44%) (Fig. 1).

**Conclusion:**

This survey identified key needs among school health staff, highlighting demand for support in outbreak management, airborne transmission prevention, and accessible educational materials.

**Disclosures:**

M. Salman Ashraf, MBBS, Merck & Co. Inc: Grant/Research Support

